# Glutathione S-transferase mu 1 (*GSTM1*) and theta 1 (*GSTT1*) genetic polymorphisms and atopic asthma in children from Southeastern Brazil

**DOI:** 10.1590/S1415-47572010000300007

**Published:** 2010-09-01

**Authors:** Carmen Silvia Passos Lima, Iramaia Angélica Néri, Gustavo Jacob Lourenço, Isabel Cristina Jacinto Faria, José Dirceu Ribeiro, Carmen Silvia Bertuzzo

**Affiliations:** 1 Departamento de Clínica Médica, Faculdade de Ciências Médicas, Universidade Estadual de Campinas, Campinas, SPBrazil; 2 Departament de Genética, Faculdade de Ciências Médicas, Universidade Estadual de Campinas, Campinas, SPBrazil; 3 Departamento de Pediatria, Faculdade de Ciências Médicas, Universidade Estadual de Campinas, Campinas, SPBrazil

**Keywords:** Atopic asthma, pathogenesis, *GSTM1 gene*, *GSTT1* gene

## Abstract

Xenobiotics can trigger degranulation of eosinophils and mast cells. In this process, the cells release several substances leading to bronchial hyperactivity, the main feature of atopic asthma (AA). *GSTM1* and *GSTT1* genes encode enzymes involved in the inactivation of these compounds. Both genes are polymorphic in humans and have a null variant genotype in which both the gene and corresponding enzyme are absent. An increased risk for disease in individuals with the null GST genotypes is therefore, but this issue is controversial. The aim of this study was to investigate the influence of the *GSTM1* and *GSTT1* genotypes on the occurrence of AA, as well as on its clinical manifestations. Genomic DNA from 86 patients and 258 controls was analyzed by polymerase chain reaction. The frequency of the *GSTM1* null genotype in patients was higher than that found in controls (60.5% *versus* 40.3%, p = 0.002). In individuals with the *GSTM1* null genotype the risk of manifested AA was 2.3-fold higher (95%CI: 1.4-3.7) than for others. In contrast, similar frequencies of *GSTT1* null and combined *GSTM1* plus *GSTT1* null genotypes were seen in both groups. No differences in genotype frequencies were perceived in patients stratified by age, gender, ethnic origin, and severity of the disease. These results suggest that the inherited absence of the *GSTM1* metabolic pathway may alter the risk of AA in southeastern Brazilian children, although this must be confirmed by further studies with a larger cohort of patients and age-matched controls from the distinct regions of the country.

Atopic asthma (AA), a disease caused by a combination of genetic and environmental factors, is characterized by a broad variety of clinical manifestations these ranging from increased bronchial susceptibility in healthy individuals to a lethal form.

Previous studies have shown that a great variety of xenobiotics can trigger degranulation of eosinophils and mast cells, with the subsequent release of various substances, active in bronchial inflammation and spasms, the main events in pathogenesis of the disease (Demoly *et al.*, 1997). Enzymes of the glutathione S-transferase (GST) system protect the bronchii from the effects of these agents by transforming them into less active compounds (Demoly *et al.*, 1997; Scoggan *et al.*, 1997; Hayes *et al.*, 2005). *GSTM1* and *GSTT1* genes of the GST system are polymorphic and homozygous null in about 40%-60% and 10%-20% of individuals of distinct ethnic origin (Hayes and Pulford, 1995; Gattás *et al.*, 2004; Hayes *et al.*, 2005), resulting in a lack of the active proteins. Apparently, people with a reduced ability in detoxification or catabolism of inductors of bronchial inflammation are under an increased risk for AA (Ketley *et al.*, 1975; Scoggan *et al.*, 1997). However, the association of GST null genotypes with this risk is controversial (Ivaschenko *et al.*, 2001; Freidin *et al.*, 2002; Brasch-Andersen *et al.*, 2004; Saadat *et al.*, 2004; Tamer *et al.*, 2004; Ercan *et al.*, 2006; Holla *et al.*, 2006; Hanene *et al.*, 2007; Mak *et al.*, 2007; Salam *et al.*, 2007; Imboden *et al.*, 2008; Islam *et al.*, 2009; Castro-Giner *et al.*, 2009; Rogers *et al.*, 2009).

The high prevalence of AA (Chatkin *et al.*, 2003) and environmentally-related diseases (Ruiz *et al.*, 1994) in southeastern Brazil has already been described. Thus, the identification of GST genotypes in AA patients and controls from this part of the country was considered essential for testing their influence in the risk and on clinical manifestations of the disease in the population as a whole.

We analyzed 86 consecutive AA patients (median age 10 years and range 7-17; 39 males and 47 females; 79 Caucasians and 7 Afro-Americans) seen at the University Hospital of the State University of Campinas, from July, 2005 to January, 2006. A background history of recurrent and reversible symptoms of air passage obstruction, high serum IgE levels, positive test results to at least one of the recognised allergens inoculated in skin, and a family history of allergy were required for AA diagnosis, in accordance with current Global Initiative for Asthma (GINA) criteria (GINA Report, Global Strategy for Asthma Management and Prevention, 2009). None of the patients enrolled in this study had any identifiable alcohol use or smoking history. All the patients were students and were classified as having a mild, moderate or severe form of AA, as previously described by Warner *et al.* (1998). The control group consisted of 258 healthy blood donors (median age 53 years and range 25-62; 117 males and 141 females; 237 Caucasians and 21 Afro-Americans) from the same University Hospital, who were matched against the controls by gender and ethnic origin, thereby providing a representative group of the general population that seeks medical assistance in this region. Both patients and controls were classified as Caucasians or Afro-Americans based on individual phenotype. The study was approved by the local review board guidelines, and all subjects gave written and informed consent in accordance with the Helsinki Declaration.

Genomic DNA was obtained from the peripheral blood of all subjects. *GSTM1* and *GSTT1* genes were amplified by multiplex polymerase chain reaction (PCR) in the same reaction, including the amplification of a β-globin gene fragment used as a control of the DNA sample (Arruda *et al.*, 2001). Genotypes were analyzed by electrophoresis on a 2.0% agarose gel, and only those PCR signals were considered in which the corresponding β-globin gene internal control was evident.

Statistical significance of the differences between groups was calculated by the chi-square or Fisher exact tests. Crude odds ratios (ORs) were calculated and are given with 95% confidence intervals (CI). Logistic regression analysis was used to obtain gender and ethnic origin-adjusted ORs. Factors with p < 0.05 were considered statistically significant. All analyses were undertaken using the statistical package SAS System for Windows, version 8.1 (SAS Institute Incorporation, USA).

A high prevalence of the *GSTM1* null genotype was seen in our AA patients. Individuals with the *GSTM1* null genotype had a 2.3-fold increased risk for manifested disease than others. No differences in frequencies of the *GSTT1* null and *GSTM1* plus *GSTT1* null genotypes were found in patients and controls. Individuals with distinct genotypes incurred the same AA (Table 1). Similar frequencies of the *GSTM1* null, *GSTT1* null and *GSTM1* plus *GSTT1* null genotypes were seen in patients stratified by age at diagnosis, gender, race, and severity of the disease (Table 2).

Upon investigating the *GSTM1* and *GSTT1* genotype frequencies of the GST system in Brazilian individuals, we found that the *GSTM1* null genotype was associated with an increased risk for AA. There are several explanations for the role of GSTs in disease pathogenesis, the first being that xenobiotics are not discharged from the organism in the absence of these enzymes, and therefore may trigger mast cell degranulation (Demoly *et a*l., 1997; Hayes *et al.*, 2005). Secondly, it is known that leukotrienes released by mast cells and eosinophils, important compounds in bronchial constriction, are inactivated by GSTs (Scoggan *et al.*, 1997). Thus, persistent bronchial spasms may be expected in the absence of enzymes encoded by the *GSTM1* and *GSTT1* genes. A further explanation is that GSTs are capable of binding steroid hormones, which are inhibitors of eosinophils involved in a late stage of the allergic reaction, and to transport them to tissues (Ketley *et al.*, 1975). The absence of these enzymes may, therefore, contribute to asthma by abnormalities in the transport of inflammatory inhibitors to bronchioles. Taken together, these observations suggest that GSTs act at different stages in the formation of an asthmatic reaction.

The results of 14 epidemiological studies on the role of GST genotypes in asthma, undertaken in countries of the northern hemisphere, were controversial. The *GSTM1* null genotype was associated with an increased risk for disease in some reports (Ivaschenko *et al.*, 2001; Brasch-Andersen *et al.*, 2004; Saadat *et al.*, 2004; Tamer *et a*l., 2004; Hanene *et al.*, 2007; Islam *et al.*, 2009; Rogers *et al.*, 2009), but not in others (Freidin *et al.*, 2002; Ercan *et al.*, 2006; Holla *et al.*, 2006; Mak *et al.*, 2007; Salam *et al.*, 2007; Imboden *et al.*, 2008; Castro-Giner *et al.*, 2009). Similarly, an increased risk was seen in individuals with the GSTT1 null genotype in some studies (Ivaschenko *et al.*, 2001; Brasch-Andersen *et al.*, 2004; Saadat *et al.*, 2004; Tamer *et al.*, 2004; Hanene *et al.*., 2007), whereas no association of the disease with this genotype was noted by others (Freidin *et al.*, 2002; Ercan *et al.*, 2006; Holla *et al.*, 2006; Mak *et al.*, 2007; Salam *et al.*, 2007; Imboden *et al.*, 2008; Islam *et al.*, 2009; Castro-Giner *et al.*, 2009; Rogers *et al.*, 2009). Differences in gene frequencies among various ethnic groups, as well as exposure to environmental chemical agents in the various countries, may explain the differences encountered. The ethnic origin of the Brazilian population is highly heterogeneous (Gattás *et al.*, 2004), consisting of indigenous Amerindians and immigrants from Europe, Africa and Asia whilst populations in the northern hemisphere are usually characterised by ethnical homogeneity. In fact, the frequencies of *GSTM1* and *GSTT1* null genotypes in controls varied from 40.8% (Turkey) to 73.5% (Russia) and 22.1% (Czech Republic) to 53.3% (China), respectively, among individuals of distinct populations in consistent studies (Hayes *et al.*, 2005). On the other hand, Brazilian workers are frequently exposed to numerous chemical agents, such as, hexachlorobenzene, carbon tetrachloride, perchloroethylene, solvents, benzopyrene, alachlor, atrazine, lindane and methyl parathion, some of which are known to be metabolised by the enzymes of the GST system (Hayes *et al.*, 2005), besides being involved in bronchial reactivity (Demoly *et al.*, 1997). Unfortunately there was a lack of reliable data focusing individual chemical exposure on the part of our patients and controls, as well as those enrolled in other studies. All the enrolled AA patients were childrens, and therefore apparently had no history of excessive exposure to chemical agents. However, we can not exclude chemical and tobacco exposition in our controls, who were older than our patients.

We also analyzed GST genotypes in clinical features of AA patients, and found no role for *GSTM1* and *GST1* by age at diagnosis, gender, ethnic origin and severity of the disease. Freidin *et al.* (2002) and Ercan *et al.* (2006) also found no association of GST genotypes with severity of the disease. On the contrary, Carrol *et al.* (2005) and Holla *et al.* (2006) found an excess of *GSTM1* null genotype in patients with malfunction of the lung, thereby implying that a lack of the M1 enzyme could contribute to lung damage in patients with the disease.

In summary, our results are suggestive that the inherited absence of the *GSTM1* metabolic pathway may alter the risk for manifested AA in our region, even though no influence on the severity of the disease was detected. However, these results must be confirmed by further studies with a larger cohort of patients and age-matched controls from distinct regions of the country.

## Figures and Tables

**Table 1:** *GSTM1* and *GSTT1* genotypes in atopic asthma patients and controls.

	*GSTM1*			*GSTT1*			*GSTM1/T1*		
	Present n (%)	Null n (%)		Present n (%)	Null n (%)		Both present n (%)	One null n (%)	Both null n (%)
Patients	34 (39.5)	52 (60.5)		72 (83.7)	14 (16.3)		27 (31.4)	52 (60.5)	7 (8.1)
Controls	154 (59.7)	104 (40.3)		213 (82.6)	45 (17.4)		130 (50.4)	107 (41.5)	21 (8.1)
OR (CI 95%)	1.0 (ref)	2.3 (1.3-3.3)		1.0 (ref)	0.9 (0.5-1.7)		1.0 (ref)	2.3 (1.4-4.0)	1.6 (0.6-4.1)
p value	0.002			0.870				0.002	0.425

OR: odds ratio; CI: confidence interval.

**Table 2:** *GSTM1 and T1* genotypes in atopic asthma patients stratified by pertinent characteristics.

Clinical features	n	*GSTM1*			*GSTT1*			*GSTM1/T1*		
		Present n (%)	Null n (%)		Present n (%)	Null n (%)		Both present n (%)	One null n (%)	Both null n (%)
Median age at diagnosis	86									
≤ 10 years	51	23 (26.8)	28 (32.5)		42 (48.8)	9 (10.5)		19 (22.1)	27 (31.4)	5 (5.8)
> 10 years	35	11 (12.8)	24 (27.9)		30 (34.9)	5 (5.8)		8 (9.3)	25 (29.1)	2 (2.3)
p value		0.30				0.90		reference	0.18	0.96
Gender	86									
Male	39	16 (18.7)	23 (26.7)		35 (40.7)	4 (4.7)		13 (15.2)	25 (29.0)	1 (1.2)
Female	47	18 (20.9)	29 (33.7)		37 (43.0)	10 (11.6)		14 (16.2)	27 (31.4)	6 (7.0)
p value		0.97			0.28			reference	0.99	0.23
Ethnic origin	86									
Caucasian	79	30 (34.9)	49 (56.9)		66 (76.7)	13 (15.1)		23 (26.8)	50 (58.0)	6 (7.0)
Afro-American	7	4 (4.7)	3 (3.5)		6 (7.0)	1 (1.2)		4 (4.7)	2 (2.3)	1 (1.2)
p value		0.55				0.88		reference	0.19	0.97
Severity of disease	86									
Mild	28	13 (15.2)	15 (17.4)		22 (25.6)	6 (7.0)		9 (10.5)	17 (19.7)	2 (2.3)
Moderate	21	6 (7.0)	15 (17.4)		19 (22.1)	2 (2.3)		6 (7.0)	13 (15.2)	2 (2.3)
Severe	37	15 (17.4)	22 (25.6)		31 (36.0)	6 (7.0)		12 (13.9)	22 (25.6)	3 (3.5)
p value		0.44			0.53			reference	0.96	0.93
